# Pollen specialist bee species are accurately predicted from visitation, occurrence and phylogenetic data

**DOI:** 10.1007/s00442-024-05653-5

**Published:** 2024-12-18

**Authors:** Colleen Smith, Nick Bachelder, Avery L. Russell, Vanessa Morales, Abilene R. Mosher, Katja C. Seltmann

**Affiliations:** 1 Cheadle Center for Biodiversity and Ecological Restoration, University of California, Harder South Building 578, Santa Barbara, CA 93106 USA; 2 Department of Biology, Missouri State University, 910 S John Q Hammons Parkway, Temple Hall, Springfield, MO 65897 USA

**Keywords:** Diet breadth, Generalist, Bee, Oligolecty, Interactions, Pollen

## Abstract

An animal’s diet breadth is a central aspect of its life history, yet the factors determining why some species have narrow dietary breadths (specialists) and others have broad dietary breadths (generalists) remain poorly understood. This challenge is pronounced in herbivorous insects due to incomplete host plant data across many taxa and regions. Here, we develop and validate machine learning models to predict pollen diet breadth in bees, using a bee phylogeny and occurrence data for 682 bee species native to the United States, aiming to better understand key drivers. We found that pollen specialist bees made an average of 72.9% of their visits to host plants and could be predicted with high accuracy (mean 94%). Our models predicted generalist bee species, which made up a minority of the species in our dataset, with lower accuracy (mean 70%). The models tested on spatially and phylogenetically blocked data revealed that the most informative predictors of diet breadth are plant phylogenetic diversity, bee species’ geographic range, and regional abundance. Our findings also confirm that range size is predictive of diet breadth and that both male and female specialist bees mostly visit their host plants. Overall, our results suggest we can use visitation data to predict specialist bee species in regions and for taxonomic groups where diet breadth is unknown, though predicting generalists may be more challenging. These methods can thus enhance our understanding of plant-pollinator interactions, leading to improved conservation outcomes and a better understanding of the pollination services bees provide.

**Supplementary Information:**

The online version contains supplementary material available at 10.1007/s00442-024-05653-5.

## Introduction

Diet breadth affects many aspects of animal ecology and evolution. The animals with narrow diet breadths, known as dietary specialists, tend to be rarer (Slatyer et al. 2013), are less likely to be targeted by predators and more likely to evolve defense mechanisms against them (Singer et al. 2014), and may be more vulnerable to anthropogenic change (Clavel et al. 2011). While host plant specialization is common among herbivorous insects such as bees (Wood et al. 2023; Forister et al. 2015), the key factors that predict diet breadth remain uncertain (Hardy and Otto 2014). Although diet breadth for many bee species is unavailable, identifying pollen specialist and generalist bees, and associated phylogenetic, phenological, and geographic characteristics is important in understanding current bee declines (Bommarco et al. 2010) and in strategically monitoring and planting pollen host plants (Winfree et al. 2011; Payne et al. 2024).

Given that closely related insect species often exhibit similar patterns of host plant use, phylogeny may be a key driver of diet breadth. However, the evidence is mixed. For example, Slove and Janz 2011 found that while use of host plant use is highly conserved in Lepidoptera, diet breadth is not. Even still, there is some effect of phylogeny in predicting diet breadth in Lepidoptera (Slove and Janz 2011). Similar patterns are observed in bees, but the results are also mixed (Slattery et al. 2023). In social lineages, such as bumble bees, there is a trend toward generalization (Wood et al. 2023), although the specific host plant use is dissimilar across bumble bee species (Wood 2021). In contrast, diet breath can transition from specialist to generalist within genera, as seen in *Diadasia* (Stipes and Tepedino 2005), and specialization may occur with different plant families within the same genus, as observed in *Osmia* (Sedivy et al. 2013). Thus, both bee and plant phylogeny may be important predictors of diet breadth.

The biogeographical factors, such as geographic location and range size may also be predictive of bee diet breadth. In general, specialization in insect herbivore diet breadth increases at lower latitudes (Schemske 2009). In contrast, bee specialization may be highest in seasonally dry, mid-latitudes regions (Wood 2023), which is also where bees are most speciose. Range size is also often strongly associated with diet breadth, with larger range size leading to broader diet breadths in arthropods (Slatyer et al. 2013). For instance, broader range size is associated with increased diet breadth in gall-inducing sawflies (Tenthredinidae: *Euurina*) and their parasitoids (Galiana et al. 2023), and diet breadth increases with latitudinal range size in Lepidoptera (Gaston 1988; Seifert and Fiedler 2024). Although there is no directly comparable study in bees, eusocial bees are known to have wider diet breadths than solitary bees (Wood 2023), and broader foraging ranges (Kendall et al. 2022), which may be linked to the ability to expand their ecological niches (Lancaster 2022) and may lead to range expansion.

Finally, phenology may also play a role in predicting diet breadth in bees. For instance, Anderson et al. (2021) found that the phenology of *Hesperapis regularis* (Melittidae), a specialist on the plant *Clarkia*, closely aligns with *Clarkia’*s flowering period, whereas *Lasioglossum incompletum* (Halictidae), a generalist *Clarkia* pollinator, does not show this alignment. These patterns may be influenced by lineage and geographic-specific traits. For instance, *Andrena*, a bee genus with a high proportion of specialists, includes some of the earliest-flying species in temperate and boreal regions. However, in general, specialist bees in temperate climates tend to become active later in the season compared to generalists (Pelletier and Forrest 2023) and have shorter periods of adult activity (Glaum et al. 2021).

A major barrier in understanding what factors drive diet breadth in bees is the shortage of diet breadth data (Dorado et al. 2011; Chacoff et al. 2012). A recent synthesis of bee pollen diet breadth found that only 860 out of 20,000 bee species globally have enough pollen data to accurately categorize their diet breadths (Wood et al. 2023). Pollen datasets are challenging to collect, and bee biologists typically identify pollen specialist and generalist bees by examining the pollen contained in bee species’ larval provisions, or in pollen scopal or corbiculate loads that bees carry back to their nests (e.g., Müller and Kuhlmann 2008; Sedivy et al. 2013; Wood and Roberts 2017). The visitation datasets are much more widely available, but less authoritative and provide records of the flowering plants that a bee species is observed visiting and typically do not confirm if the bee is collecting or carrying pollen from the visited plant.

The visitation datasets may be biased based on the collector or study objectives (Meineke and Daru 2021). These datasets are often incomplete and typically contain only a subset of the interactions occurring in the area that was sampled because data collectors will inevitably miss interactions between species (Chacoff et al. 2012). For example, even when 80% of pollinator species were sampled in a study area, 45% of the pollinators’ interactions with plants were missed; sampling 90% of interactions would require a five-fold increase in sampling effort (Chacoff et al. 2012). When many interactions go undetected, generalist species may be incorrectly classified as specialists (Blüthgen 2010; Dorado et al. 2011). This problem is especially pronounced for rare species. For instance, a species observed only once in a dataset can only be detected using a single host plant species and thus may erroneously be classified as a specialist. Most ecological datasets have many of these singletons (Novotný and Basset 2000; McGill 2003). Another issue with visitation datasets is that pollen specialists often visit non-host plants for nectar, making specialist bees appear as generalists (Neff and Danforth 1991; Pekkarinen 1997; Robertson 1925). This may be especially common in male bees, which do not collect pollen for larval provisions (Danforth et al. 2019).

Therefore, to better understand the biogeographic and ecological drivers of specialization, we created a dataset of 682 bee species of the United States that includes diet breadth, phylogenetic, phenological, and geographic information, and a large bee-plant visitation dataset (*n* = 150,880) to ask (1) How often do pollen specialist bees visit their pollen host plants? (2) Can we predict pollen specialist and generalist bees from flower visitation data? And (3) what variables are most important for predicting specialist and generalist bees: phylogenetic-, phenological- or geographic variables?

## Methods

### Authoritative diet breadth datasets

To predict pollen specialist and generalist bee species, we fit predictive models using bee species with known diet breadths for the United States from authoritative sources. This dataset contains a total of 682 species (58 genera) and represents the current knowledge of bee diet breadth at the time of this study. Throughout this study, we define dietary specialist bees (hereafter, pollen specialists or specialists) as species that consume pollen from within a single family of plants, and we define generalists (or pollen generalists) as species that consume pollen from plants in more than one family, following Robertson (1925). Our diet breadth data came from two sources. First, we used a list of pollen specialist bee species and their host plants in the eastern, central, and western United States (compiled from Fowler 2020a, b; Fowler and Droege 2020; *n* = 1072 species); this list categorizes bee species as pollen specialists using both pollen and visitation data and is vetted by expert opinion. The host plants of some bee species on this list spanned multiple plant families, and we reclassified these bee species as pollen generalists.

Second, we used a bee-pollen database we compiled ourselves using a literature survey (*n* = 1492 species). We searched the scientific literature between September 2019 and August 2022 for articles or books that reported descriptions of bees’ pollen hosts using primary pollen data or that were secondary sources synthesizing bee’s pollen hosts from primary data. Because the literature survey ended in 2022, it does not include some recent papers, including Wood et al. 2023. Here, primary pollen data are defined as the plant genera or families found in bees’ scopal/corbiculate loads or nest provisions, or, rarely, the plants that the authors observed bees pollen-foraging from. We searched Google Scholar and Web of Science using the search terms “bee,” “mono/oligo/poly,” “lecty/lege/lectic,” “pollen host plant,” “pollen host,” “pollen specialization,” “pollen diet breadth,” and “host preference,” or the comparable search terms in German, French, and Portuguese. We also searched for papers cited within the articles found using this search. For each bee species, we classified a plant taxon as its pollen host if it made up at least 5% of the total scopal/corbiculate load or nest provision, following (Cane and Sipes 2006). We also considered a plant taxon to be a pollen host if the authors of the paper observed the bee collecting pollen from that plant taxon (although such studies were a minority). We classified bee species on this list as pollen specialists if their pollen hosts came from within one family and as pollen generalists if their host plants spanned more than one plant family. Our full list of specialist and generalist bees and the sources used to classify them is provided in Appendix S2 (*n* = 1292 total bee species, 435 from the Fowler dataset, 440 from the literature dataset, and 417 species with unknown diet breadth). Because this literature survey dataset relied on a broader range of studies, occasionally a bee species classified as a specialist by the Fowler dataset was classified as a generalist by the literature survey dataset. In such cases we used the classification of the literature survey dataset, resulting in 59 replacements. Hereafter, we refer to these combined datasets as the ‘authoritative dataset.’

### Visitation dataset

The visitation data we used to predict bee species’ diet breadths came from bee species visitation records found in Global Biotic Interactions (GloBI) (https://www.globalbioticinteractions.org/; Poelen et al. 2014). GloBI is an open dataset indexer that unifies species interactions across scientific literature, specimens from natural history collections, published research and online observations. GloBI provided bee species visitation records across large spatial scales and with large sample sizes—the total dataset contains 259,210 bee visitation records between bees and plants (i.e., prior to filtering by geography or other variables, see below). We used version 0.5 of the GloBI indexed dataset (GloBI Community 2022).

We first filtered the GloBI database to only include interactions between bees and plants, searching for interactions between individuals within the seven families of bees and individuals within the kingdom Plantae. We excluded records where plants were not identified to at least the genus level and bees to the species level, as well as all duplicate records from the data. Hereafter, we refer to this dataset as the ‘visitation dataset.’ Note, this may exclude some species that have unresolved taxonomy and/or are rare—and thus harder to classify.

We further filtered the data to only include bee species that occur in the contiguous United States. Because not all records in the visitation dataset have geographic coordinates, we determined which bee species occur in the contiguous United States using a list from Chesshire et al. (2023), which was compiled using specimen records from GBIF and SCAN (GBIF.org 2021a; b, c). We then excluded cleptoparasites (Michener 2000; Appendix S2) and non-native bee species, using a list of non-native bee species for the United States (Russo 2016; Appendix S1: Table S1). We also removed records of eight bee species for which we did not have any phenological data (see ‘Estimating geographical and phenological predictors’). Finally, we excluded species we did not have diet breadth data for (Appendix S2).

We updated bee taxon names from the visitation dataset to the current valid name following the same methodology outlined in Chesshire et al. (2023); see Appendix S1 Supporting Methods for more details). We updated plant taxon names from the visitation dataset using The Plant List (www.theplantlist.org). Although this list is outdated (last updated in 2013), we opted to use it because this was the taxonomic standardization method employed by the plant phylogeny we used (Jin and Qian 2019; see next section). We accessed The Plant List using the R package *Taxonstand* (Cayuela et al. 2012) and updated plant family names separately, using the World Flora Online (http://www.worldfloraonline.org/). Plant taxa not on The Plant List were also updated using the World Flora Online. We used the same methods to update plant names in the authoritative dataset. There were some cases in which there was taxonomic uncertainty regarding the families of a specialist bee species’ pollen host plant. For example, the authoritative dataset (derived from Fowler 2020a, b) listed a bee species as a specialist on plants on Capparaceae, but World Flora Online (WFO) classified the plants in both Capparaceae and Cleomaceae. We considered these bee species to have plants from both families as its host plants.

After these filtering steps and taxon name updates, our visitation dataset contained 150,880 records of 682 bee species visiting 1185 plant genera: 50,858 were records of pollen specialist bees, from 477 bee species, and 100,022 were records of generalist bees from 205 bee species. The records came from 40 sources total (Appendix S1: Table S2), with 22.6% of the records coming from observations, such as iNaturalist, 65.3% of the records coming from museum specimens (typically the flowering plant species the specimen was collected from), and 12.1% of the records coming from the scientific literature or unknown, compiled, sources (note, it is possible that some records in the “unknown” category may contain pollen data).

### Bee and plant phylogenies

We used the genus-level bee phylogeny from (Henríquez-Piskulich et al. 2024). All the genera in our dataset were present in the phylogeny. We updated five generic names in our datasets to align them with the taxonomic concepts used in the phylogeny (Appendix S1: Table S3).

To make a phylogeny of plants, we used the megatree from Smith and Brown (2018), which we accessed using the R package *V.PhyloMaker* (Jin and Qian 2019). We pruned the tree to be a genus-level phylogeny. This phylogeny was missing 103 of the plant genera in our visitation dataset, of 1185 plant genera total, and we added them randomly within their family using scenario 2 from *V.PhyloMaker* (Jin and Qian 2019). According to (Jin and Qian 2022), V.PhyloMaker has been used in 217 published studies according to Thompson Reuters ISI Web of Science (access on April 21, 2022). The authors also investigated how different tree construction methods affect the quantification of phylogenetic distance. They found that the relationship between phylogenetic metrics and environmental variables generally remains consistent, whether using a phylogeny resolved at the species level or one created by linking genera to a well-resolved family-level phylogeny (Qian and Jin 2016).

### Occurrence dataset

To obtain geographic and phenological predictors for all bee species in our interaction dataset, we used specimen records from North America (Chesshire et al. 2023). This dataset included specimens in Canada, Mexico, and Alaska. To ensure independence of the specimen records, we filtered the dataset to have one record of each species per combination of latitude, longitude, and collection date. The latitude and longitude coordinates were rounded to three decimal places prior to this filtering step. We removed geographic outliers, defined as specimens collected at least 1500-km from any other specimen of the same species. Hereafter, we refer to this as the Chesshire et al. (2023) occurrence dataset.

### Estimating visitation predictors

We used the visitation dataset to identify both the plant taxa a bee species visits and the number of taxa it visits. To quantitatively measure which plant taxa a bee species visits, we used a multivariate approach: we first built a matrix with bee species as rows and plant genera as columns, with matrix cells filled with the number of interactions observed. We used the matrix and the Morisita-Horn index to calculate the difference between each pair of bee species in the plant genera visited. We took the first two eigenvectors of the resulting distance matrix to use in our models to get an eigenvalue for each bee species. Similar eigenvalues indicate bee species visit similar plant taxa. We also estimated these eigenvalues for interactions between bee species and plants at the plant-family level.

To estimate how many plant taxa each bee species visits, we calculated the diversity of plant genera and families visited using the inverse Simpson index. The diversity of plant families a bee visited was strongly correlated with phylogenetic diversity of plant genera it visited (*r* > 0.7) and the Simpson diversity of plant genera visited (r > 0.7). We thus excluded this variable (plant families a bee visited) from our final model.

Finally, we estimated the phylogenetic diversity of plant genera a bee species visits based on our reconstructed bee and plant phylogenetic trees. In contrast to taxonomic diversity, phylogenetic diversity will be higher for bee species that visit distantly related plant genera than for bee species that visit closely related plant genera. We estimated phylogenetic diversity using a phylogenetic generalization of the inverse Simpson index (Chao et al. 2010, 2014), and hereafter refer to this metric as “phylogenetic Simpson diversity.” We also estimated phylogenetic richness. However, this variable was strongly correlated with a number of other variables in the model (*r* > 0.7). We estimated phylogenetic Simpson diversity using the function ‘hill_phylo’ from the R package *hillR* (Li 2018). More details about how we calculated this metric are provided in the Supporting Methods (Appendix S1).

### Estimating bee phylogenetic predictors

We included bee phylogenetic information in our model, following the approach used in Lucas (2020). For each bee species, we calculated the phylogenetic distance to each bee genus in the dataset and used the resulting distances as predictor variables. Thus, for each bee species, there were 58 phylogenetic predictor variables, one for each bee genus. We used the function ‘cophenetic’ from the package *ape* to calculate pairwise phylogenetic distance between bee genera (Paradis and Schliep 2019).

### Estimating geographic and phenological predictors

To estimate each bee species’ approximate geographic range, we first calculated a central point using the median latitude and longitude of the specimen records in the Chesshire et al. 2023 occurrence dataset. To estimate each bee species’ extent of occurrence, we created a minimum convex polygon from all records and calculated the area in hectares. For species with fewer than four unique latitude–longitude combinations, there were too few points to create a minimum convex polygon. For these, we randomly added points within 100-km of existing specimen records to reach the four points needed to create the minimum convex polygon. We also calculated the sample size of each bee species in the dataset, a measure of the bee species’ regional abundance. Although the occurrence records used to estimate abundance may overestimate the relative abundance of rare species, (Gotelli et al. 2023) found a strong correlation between relative abundance calculated from occurrence records and that estimated from standardized field surveys. The geographic analyses were conducted using the R packages *sf* (Pebesma 2018) and *sp* (Pebesma and Bivand 2005; Bivand et al. 2013). The minimum convex polygons were created using the function ‘chull’ from the R package *grDevices*.

To calculate phenological predictors, we excluded specimen records without collection dates (12.4% of records). We estimated the approximate time of year the bee was active by calculating the median date of collection. To estimate the length of the bee’s flight period, we subtracted the beginning of the bee species’ activity period from the end of its activity period by subtracting the 10th percentile of the bee’s collection dates from the 90th percentile, following Harrison et al. (2019).

### Analyses

All analyses were conducted in R version 4.2.1 (R Core Team 2022) and R version 4.3.0 (R Core Team 2023). The code and data for running the analyses are available on Zenodo: 10.5281/zenodo.8347145.

#### How often do pollen specialist bees visit their pollen host plants?

To assess how often specialist bees visit their pollen host plants, we used the visitation dataset. For this analysis we excluded all records from generalist bee species. We calculated the proportion of times a specialist bee species was visiting its host plant out of all visits recorded. For this analysis, we excluded bee species with fewer than 20 records, to avoid assessing the visitation records of incompletely sampled species. This left us with a sample size of 300 specialist bee species and 49,710 records.

We also conducted two post-hoc analyses using the dataset of 300 specialist bees to examine why some pollen specialist species were predominantly recorded visiting non-host plants in the visitation dataset. First, we investigated if this was a statistical artifact driven by bees with small sample sizes, which are less likely to be representative of their true population. To do this, we visually examined the relationship between a bee species’ sample size and the proportion of visits to its host plant. We also calculated the Pearson correlation coefficient of this relationship.

Second, we examined whether the pollen specialist bees predominantly recorded visiting non-host plants could be explained by the presence of male bees in the visitation dataset. Male bees do not collect pollen for their offspring and, as a result, might nectar at non-host plants more frequently (Roswell et al. 2019). We opted not to initially filter the visitation dataset exclusively to female bees because the sex of the bee was only specified in 58% of the records in our visitation dataset. For this test, we narrowed down our analysis to bee species with at least 10 records each for male and female bees, resulting in 260 specialist bee species from 34,822 records. We then compared the percentage of visits made by male and female bees to their pollen hosts using a paired Wilcoxon signed ranks test.

#### Can we predict pollen specialist and generalist bees from flower visitation data?

To predict whether a bee species is a specialist or generalist, we used a random forest model for binary classification, using the R package *randomForest* (Liaw and Wiener 2002). The random forests are a type of supervised machine learning, which make no distributional assumptions and can detect complex, non-linear relationships. In our random forest, we used the default parameters from the R package: decision trees were created using bootstrapped samples the same size as the data, and ten random predictor variables were considered at each tree split. The decision trees were optimized by finding the tree with the smallest node impurity. The full set of predictor variables used in our random forest model are described in Table 1.

**Table 1:** Predictor variables considered in the random forest models to predict bee diet breadth. We removed some predictors due to collinearity (Pearson correlation coefficient > 0.7). Variables we excluded for this reason are indicated with a “No” in the “Included?” column

Predictor variable	Description	Included?	Dataset used
Phylogenetic richness	Faith’s phylogenetic diversity of plant genera visited	No	Global Biotic Interactions
Phylogenetic Simpson diversity	Phylogenetic Simpson diversity of plant genera visited	Yes	Global Biotic Interactions
Simpson diversity (plant genus)	Simpson diversity of plant genera visited	Yes	Global Biotic Interactions
Simpson diversity (plant family)	Simpson diversity of plant families visited	No	Global Biotic Interactions
Identity of plant genera visited	First and second eigenvalues of Morisita-Horn distance-matrix for plant genera visited	Yes	Global Biotic Interactions
Identities of plant families visited	First and second eigenvalues of Morisita-Horn distance-matrix for plant families visited	Yes	Global Biotic Interactions
Median latitude	Median latitude of bee specimen records in North America	Yes	Chesshire et al. (2023)
Median longitude	Median longitude of bee specimen records in North America	Yes	Chesshire et al. (2023)
Regional abundance	Number of specimen records in North America	Yes	Chesshire et al. (2023)
Extent of occurrence	Area in hectares of minimum convex polygon for specimen records in North America	Yes	Chesshire et al. (2023)
Median day-of-year	Median day-of-year of collection	Yes	Chesshire et al. (2023)
Duration of flight season	90% quantile of day-of-year of collection—10% quantile of day-of-year of collection	Yes	Chesshire et al. (2023)
Pairwise phylogenetic distance	Phylogenetic distance to each bee genus in the dataset	Yes	Henríquez-Piskulich et al. (2024)

To assess model performance, we used k-fold cross validation, in which separate datasets are used to train and test the model. In this process, the data are divided into eight k folds: k-1 folds are used to train the model and the remaining fold is used to test the model. This is repeated until all k folds have been used to test the model.

We used k-fold cross-validation to evaluate the effectiveness of our model in predicting specialist bees across different geographic regions and phylogenetic groups. While dividing our data into training and testing sets, we used spatial and phylogenetic blocking (Bahn and McGill 2013; Roberts et al. 2017). This approach leads to the creation of datasets that are either spatially or phylogenetically independent. It provides more accurate assessment of predictive power than the conventional random selection of folds (Bahn and McGill 2013; Roberts et al. 2017). By using this technique, we can assess how well our model performs when dealing with bee species located in different regions or originating from distinct families compared to those used to train the model. As a baseline, we also used random-stratified blocking to see how blocking methods affected our results.

For phylogenetic blocking, we blocked bees by family. However, the smallest family in our dataset, Melittidae, had only three generalist bee species. We, therefore, combined this family with Colletidae, the second smallest bee family in our dataset. Thus, we grouped by sample size rather than by phylogenetic distance. Given that all extant evolutionary cousins in phylogenetic trees are equally related to shared common ancestors (Gregory 2008) and Melittidae’s basal position in Apoidea (Danforth et al. 2013), we chose to combine them.

For the spatial blocking methods, we removed all spatial predictors from the models. For the phylogenetic blocking methods, we removed all phylogenetic predictors from the models. We did this to avoid extrapolating outside the predictor space used to train the model. The three blocking methods (random, spatial, and phylogenetic) are described in more detail in the Supporting Methods (Appendix S1).

We used the same metrics to assess model performance for all blocking methods. As measures of overall model performance, we used the area under the receiver operator curve (AUC) and balanced accuracy (the arithmetic mean of specialist and generalist prediction accuracies); both are insensitive to class imbalance, which we had in our dataset (70% specialist species and 30% generalist species). We also calculated the prediction accuracies of specialists and generalists.

We found that model performance was similar between random-stratified blocking and the other two blocking methods (see Appendix S1: Figure S1). We report model performance metrics for spatial and phylogenetic blocking methods as they compare to random-stratified blocking and report the metrics for all three blocking methods in Appendix S1 (Figure S1).

We also conducted a comparison between the random forest models and a simpler phylogenetic model. In this simpler model, we predicted that the diet breadth of a bee species was the same as the diet breadth of the majority of bee species within its genus, based on the training data. For bee species with no congeners in the training data, we predicted its diet breadth to be the same as the majority of bee species within its family. We evaluated the performance of this simpler model using spatial cross validation, employing the same methods as for the random forest model.

#### What variables are most important for predicting specialist and generalist bees?

To determine what variables are important for predicting specialist and generalist bees we used the “importance” function in the package *randomForest* to calculate each variable’s importance. The function calculates the change in the error rate of the model when a predictor variable is permuted, divided by the standard deviation of the difference. To rank the importance values, we took the mean of the importance values for each predictor. The means were calculated by aggregating across all model runs from all blocking methods. We also assessed the importance of phylogenetic predictors in aggregate by removing them from spatially blocked models and calculating the change in the models’ mean accuracy and AUC.

## Results

### How often do pollen specialist bees visit their pollen host plants?

**Fig. 1:**
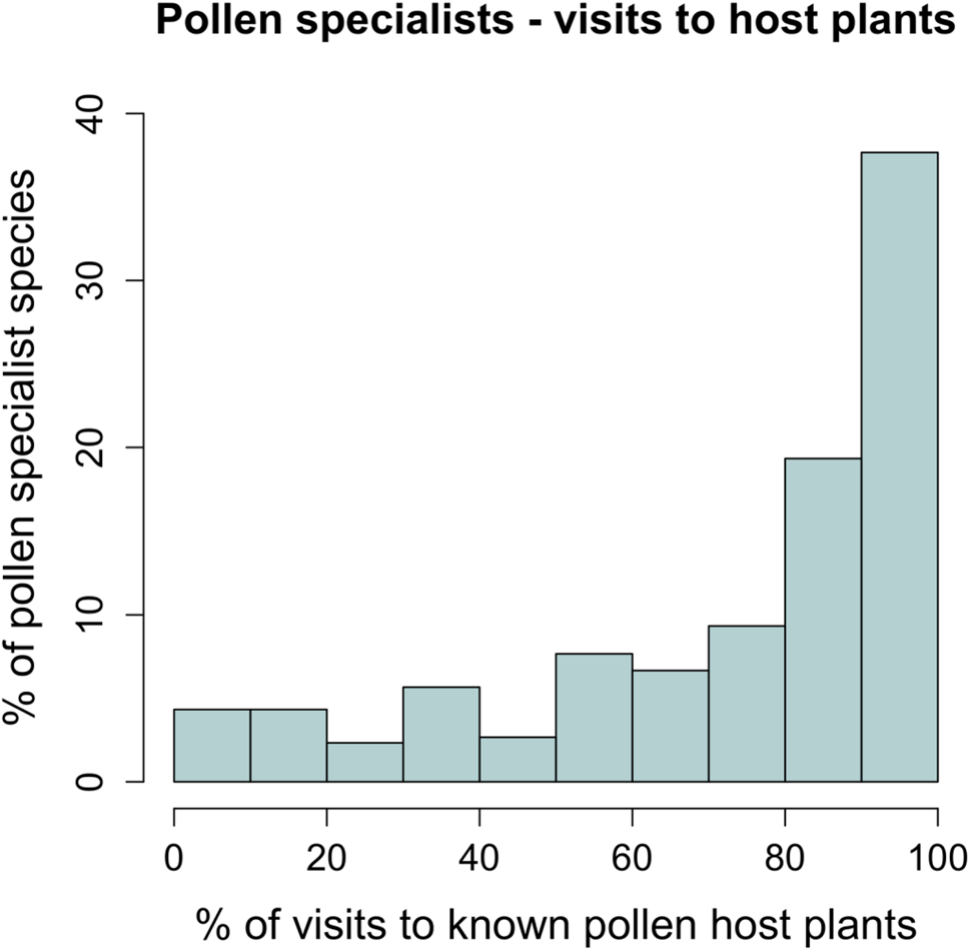
Histogram depicting how frequently specialist bees visit their known pollen host plants in the visitation dataset. Only bee species with at least 20 observations were included. Only 5% of bee species (*n* = 15 species) visited their known pollen host plants less than 10% of the time; 38% (*n* = 114) bee species visited their known pollen host plants at least 90% of the time

In our visitation dataset, we found that on average 72.9% of the visits made by pollen specialist bees were to their host plants (median = 82.9%; Fig. 1), with approximately 10% of specialist bees having 100% of visitation records to their host plant. For five specialist bee species, there were no records of the bee visiting their pollen hosts. These species were *Dufourea virgata* (Cockerell, 1898), *Megachile frigida* (Smith 1853), *Perdita layiae* (Cockerell, 1938), *Svastra sila* (LaBerge, 1956), and *Svastra atripes* (Cresson, 1872).

**Fig. 2:**
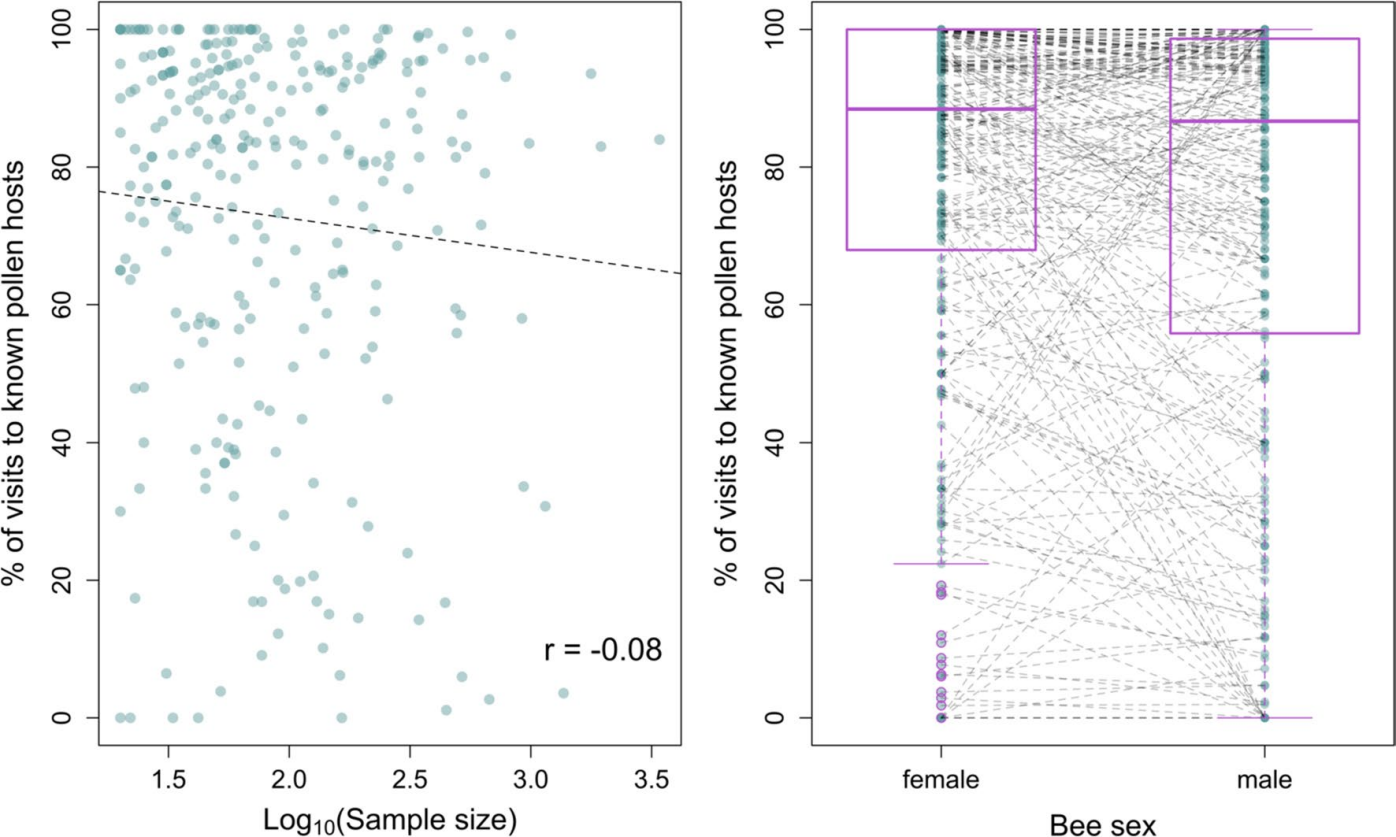
**A** The relationship between a specialist bee species’ sample size and the percentage of times the bee species visits its known pollen host in the visitation dataset. **B** Boxplots showing the percentage of times male and female bees of specialist species visit their host plants. Dotted lines connect males and females of the same species. The points represent the data. The boxes encompass the first and third quartiles of the data and the thick black line is the median. Plot whiskers extend to 1.5 times the interquartile range and the black outlined circles represent outliers

Many pollen specialist bee species that mostly visited non-host plants had smaller sample sizes (see bottom left cluster of points in Fig. 2a), suggesting these bees may be a statistical artifact. Overall, the relationship between host plant fidelity and sample size was weakly negative (*r* = −0.08) and there were common bee species visiting their host plants less than 50% of the time. Eleven species had over 200 records with less than half to the putative pollen host, including *Protoxea gloriosa* (Fox, 1983; *n* = 1366, 3% of visits to its pollen host), *Megachile brevis* (Say, 1837; *n* = 1148, 30% of visits to its pollen host) and *Megachile mendica* (Cresson, 1878; *n* = 934, 33% of visits to its pollen host; see Appendix S1: Table S4 for full list and host plants).

Male bees were also significantly more likely to visit nonhost plants than female bees of the same species (Wilcoxon signed ranks test: *V* = 15,666*; p* < 0.0001). However, the effect size was small: 77% of visits were to host plants for females vs 72% for males (Fig. 2).

### Can we predict pollen specialist and generalist bees from flower visitation data?

In our random forest models (random blocking method), we achieved 86.7% mean overall accuracy (8 percentage points better than a naïve majority guessing approach), and a mean balanced accuracy of 81.8%. Moreover, we achieved 93.9% mean accuracy at predicting specialists, a 69.7% mean accuracy at predicting generalists, and a mean AUC score of 0.90.

The blocking methods did not strongly affect overall model performance or the model’s ability to predict specialists. Models achieved a mean AUC of 0.82 and mean specialist accuracy of 91.4% when tested and trained on phylogenetically independent sets of data (phylogenetic blocking); they achieved a mean AUC of 0.84 and a mean specialist accuracy of 91.8% when tested on and trained on spatially independent sets of data (spatial blocking). However, the spatially blocked models tended to perform worse at predicting generalists (58% mean accuracy vs 66% for phylogenetically blocked models). At times, the spatially blocked models’ predictions were worse than a coin toss at predicting generalists (minimum prediction accuracy = 25%).

Our simple phylogenetic models performed well at predicting specialists (mean accuracy = 91.4%) and had moderate overall performance (mean balanced accuracy = 78.5%), but they performed less well at predicting generalists (mean accuracy = 65.6%; minimum accuracy = 52.9%). Table S5 (Appendix S1) provides a list of specialist bee species for which our random forest models provide a specialist classification probability of less than 50%. Table S6 (Appendix S1) provides the same information for generalists.

### What variables are most important for predicting specialist and generalist bees?

**Fig. 3:**
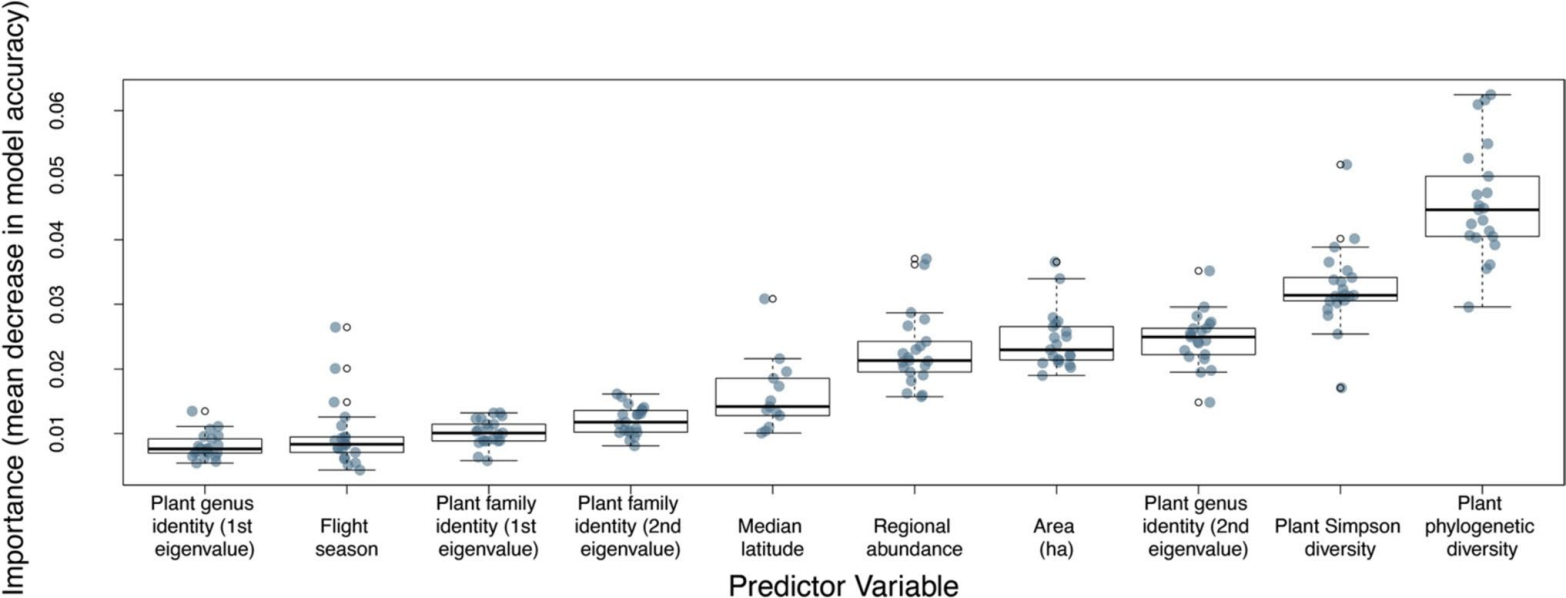
The importance values for top 10 predictor variables of pollen specialist and generalist bees. Importance is defined as the standardized decrease in the out-of-bag classification rate between the observed value and null expectation for that variable. The points show the importance value of the predictor for each model run, with a random jitter added to allow overlapping points to be seen. The boxes encompass the first and third quartiles of the data and the thick black line is the median. Plot whiskers extend to 1.5 times the interquartile range and the smaller black circles represent outliers

The two most important variables for predicting specialist and generalist bees were the phylogenetic and taxonomic diversity of plant genera visited (Fig. 3). On average, the phylogenetically blocked models predicted that a bee species had a 73.4% chance of being a specialist if it visited the smallest phylogenetic diversity of plants in the dataset vs a 56.0% if it visited the greatest (holding other covariates at their true values; Appendix S1: Figure S1). Similarly, for Simpson diversity, the phylogenetically blocked models predicted that a bee species had a 76.0% chance of being a specialist if it visited the smallest taxonomic diversity of plants in the dataset vs a 56.6% chance if it visited the greatest (Fig. 3). Other important variables for predicting bee diet breadth included the identities of the plant genera a bee visited, the bee species’ extent of occurrence, median latitude of the bee, and the bee species regional abundance (Fig. 3). Plant phenology and phylogenetic distances between bee genera were not important. The full list of mean importance values for all predictors is provided in the Supporting information (Appendix S2: Table S7).

Overall, we found that bee phylogenetic variables in aggregate had minimal effect on model performance. When bee phylogenetic predictors were removed from the spatially blocked models, overall model performance changed little (change in average AUC from 0.84 to 0.80, change in overall accuracy from 84.7% to 84.1%), as did prediction accuracy for generalists and specialists (change in generalist prediction accuracy from 57.5% to 58.2%; change in specialist prediction accuracy from 91.8% to 92.2%).

## Discussion

Our analyses reveal that pollen specialist bees visit their pollen host plants 72.9% of the time and that these species can be predicted using visitation, geographic, phenological, and phylogenetic data. The performance for predicting generalists was moderate, likely due to class imbalance in our data (30% generalists vs 70% specialists), but overall model performance was high, with AUC scores above 0.8. The random forest models performed substantially better than simpler phylogenetic models without geographic, phenological or visitation data. Below, we discuss the significance and applications of our findings.

In our study, we find that visitation data—used alongside plant phylogenetic and occurrence data—can be used to determine, with reasonable confidence, if a bee species is a pollen specialist. This conclusion is different from the one bee biologist Charles Robertson came to almost a century ago (Robertson 1925). Robertson found that, in his smaller sample (*n* = 570 records total; 253 non-host nectar visits vs 317 visits to host plants), almost half of all visits that specialist bees made were to non-host plants, leading him to conclude that visitation data cannot be used to differentiate between pollen specialists and generalists. But our much larger dataset (*n* = 150,880 records total, reduced to 49,710 pollen specialists, and 300 bee species) shows that pollen specialist bees are generally more faithful visitors to their pollen hosts than what Robertson found, with pollen specialists making about 73% of visits to their pollen hosts. Pollen generalist bee species, by contrast, visited a wide phylogenetic diversity of plants, 15.3% greater than what specialists visited (Appendix S1: Figure S3). These differences in visitation, along with phylogenetic and biogeographic differences between generalists and specialists, allowed us to predict specialist bees with an average accuracy of 94% and generalist bees with an average accuracy of 70%.

Models trained and tested on spatially and phylogenetically blocked data had comparable performance to models trained and tested on data that were blocked randomly (Appendix S1: Figure S1). This suggests that the models can generalize effectively across different bee families and geographic regions. This was the case despite strong differences between families and regions in the proportion of specialists. For example, in our dataset, pollen specialist bees comprised at least three quarters of species in the families Melittidae (85.7% of species) and Andrenidae (80.5% of species), but fewer than half of the species in Halictidae (45.1% of species). Among bee genera there was even greater skew: 21% of the bee genera in our data were comprised entirely of generalists and almost half (44.8%) were comprised entirely of specialists. Similarly, there are large differences between different regions of the United States in the proportion of specialists. The western United States, known for its high bee species diversity, hosts a greater number and proportion of specialist bee species than the eastern half of the country (Danforth et al. 2019; Fowler 2020a, b; Fowler and Droege 2020). Notably, the Chihuahua and Sonoran deserts are hotspots of specialist bee diversity, likely because these desert regions experience significant seasonal variation in rainfall that may promote the evolution of specialists (Minckley et al. 2000). By contrast, the eastern United States hosts fewer bee species overall and a smaller proportion of pollen specialists. Our data were consistent with these overall trends in bee biogeography: the northwestern- and northeastern-most regions in our dataset had the lowest proportion of specialists (45% and 44% of specialists, respectively) while the three regions in the southwest (spanning California to Texas) had the greatest proportion of specialists (94%, 90% and 90% of specialists).

There were some specialist bee species in our data that rarely visited their nominal pollen hosts, and five species that never visited them (Fig. 1). These bee species might be dominated by records of male bees, which do not provision nests with pollen, and likely make fewer trips to their pollen hosts than females. However, we found that males probably do not explain the pattern: they were less likely—but not dramatically less likely—to visit pollen hosts than conspecific females (Fig. 2b). Many of these “unfaithful” pollen specialists were species with small sample sizes and are thus probably statistical anomalies that do not reflect the populations they are drawn from (Fig. 2a). However, eleven pollen specialist species had more than 200 records with less than half those to the putative pollen host (Appendix S1: Table S4). These bee species may not be pollen specialists. Consistent with this, others have found that some species of putative pollen specialists carry large proportions of non-host pollen in their pollen loads (Michener and Rettenmeyer 1956; Ritchie et al. 2016; Smith et al. 2019).

It may also be that some putative pollen specialists are really what have been called “facultative oligoleges” (Cane and Sipes 2006). Cane and Sipes (2006) define this type of specialist bee species as one that has a strong preference for its host plant but will collect non-host pollen when its host plant is not in bloom, or to supplement the pollen of its host plant. Alternatively, putative pollen specialists may be geographic specialists, or bees that specialize in one location, but use other host plants in other parts of their range (Davis et al. 2012; Gaiarsa et al. 2022; Mesler and Carothers 2023).

**Fig. 4:**
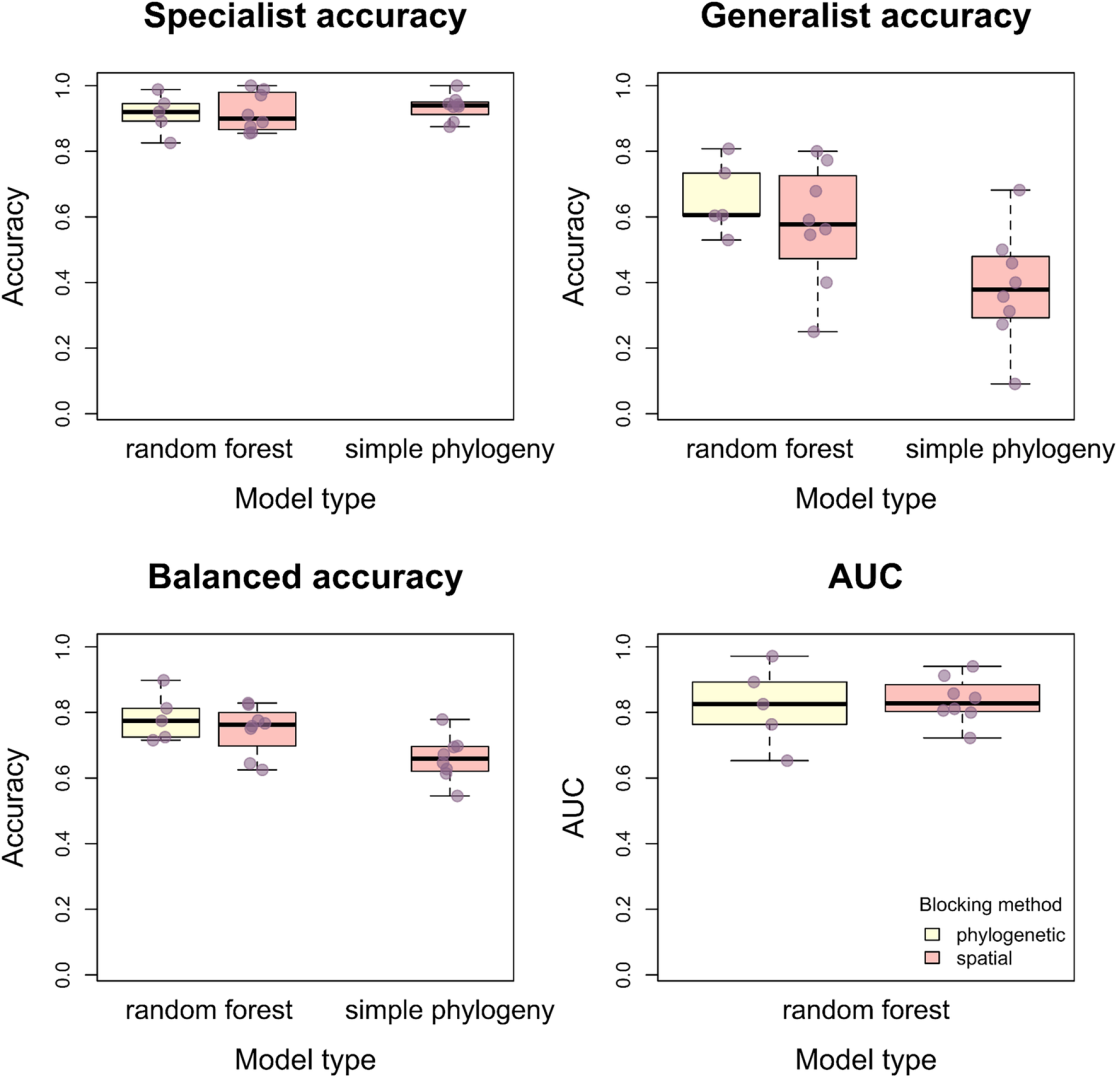
Boxplots showing estimates of model performance for the different blocking methods. Purple points represent the data, with a random jitter added. For random forest models tested using phylogenetic and spatial blocking, we excluded phylogenetic and spatial predictor variables, respectively. Thus, random forest models tested on phylogenetically blocked data lacked phylogenetic predictor variables; random forest models tested on spatially blocked data lacked spatial predictor variables. AUC was not calculated for the simple phylogenetic model

We found that generalists were more challenging for our model to predict than specialists (Fig. 4). One potential reason why is that rare generalist species are harder to classify (see Introduction). A second potential reason why is that generalist species made up the minority of bee species in our dataset. Class imbalance can affect random forest models by causing them to disproportionately favor the majority class, leading to biased predictions and poorer recognition of the minority class (Elrahman and Abraham 2013). This occurs because the model is trained on more instances of the majority class, which can result in less accurate predictions for the minority class, reducing overall model effectiveness. Although our data are not strongly imbalanced (70% of bee species in our dataset are specialists), a model fitted with our data could have an overall accuracy of 70% by predicting that bee species are specialists 100% of the time. Our models performed better than that, but future research could improve model performance by utilizing methods to explicitly deal with class imbalance (Elrahman and Abraham 2013). There is a lack of available data on the pollen carried by generalist species. This bias toward reporting the floral hosts of specialist or rare bees mirrors historical trends in museum specimen collection, where collectors often focus on uncommon species (Meineke & Daru 2021). To address this imbalance, we encourage bee biologists to report specific pollen host data for common and generalist bees as well as rare, specialist bees.

### Significance

Predicting specialist and generalist bee species is important for several reasons. First, predicting specialists allows for more targeted conservation because specialists are more strongly dependent on a limited suite of hosts to complete their life cycle. For example, planting milkweed, the larval host of the declining monarch butterfly, is seen as a critical part of this specialist’s recovery strategy (Pelton et al. 2019). For bees, diet breath information could be used to inform seed mixes used to increase bee diversity in agriculture (Morandin and Kremen 2013; Seitz et al. 2020) and urban areas (Gerner and Sargent 2022). In the United States, there are 29 specialist bee species currently rated by the website NatureServe as critically imperiled or imperiled, though the overwhelming majority (88%) of specialist bee species have not been assessed for their conservation status (www.natureserve.org; though see Bartomeus et al. (2013); Harrison et al. (2019); Lane et al. (2023) for studies that have assessed solitary bee species for their degree of rarity or relative decline). In addition to supporting species conservation, predicting specialist and generalist bee species can tell us more about the quality of pollination services provided by a bee species, with specialists potentially depleting more pollen, but also transferring more conspecific pollen than generalists (Parker et al. 2016; Smith et al. 2019).

### Applications

Our proposed modeling approach can help guide data collection on specialist and generalist bees for taxa or regions where pollen data are missing. For instance, the researchers can fit models to existing data for bee genera where lists of specialist and generalist bees have been generated using pollen data. Once the models are fit using the training data, the researchers can then use these models to predict specialists and generalists in a closely related bee genus where such lists are not available. The accuracy of the model’s predictions should improve the more closely related the bees in the training and testing data (Houlahan et al. 2017). Although our findings suggest that models phylogenetically blocked in this way will misclassify ~ 16% of species (in our data, 34% of generalists and 8% of specialists), their predictions can provide guidance for future research by pointing researchers towards the bee species most likely to be specialists or generalists. To improve future model predictions and validate its results, researchers should continue to collect pollen and publish data from the scopal loads or nest provisions of the bees (Cane and Sipes 2006), especially for generalist bees. Pollen data are essential because they reveal the plant taxa from which the bee collects pollen. This approach enables focused research efforts and directs researchers towards the bee species that are most likely to specialize.

Approximately 30% of the bees in our visitation dataset (*n* = 389 species total) were missing a specialist or generalist classification from our bee-pollen dataset and were thus excluded from our analyses. Because we had visitation, geographic and phylogenetic data for these bee species, their diet breadths might be predicted using our own modeling approach. However, the approach would need to be modified to account for the likelihood that pollen generalist bee species are over-represented in the missing data and under-represented in the training dataset. This is because we had a comprehensive list of specialist species for the United States, but not generalists (Fowler 2020a, b; Fowler and Droege 2020). In fact, generalist bees made up only 30% of the bee species in our analyzed dataset, even though they likely represent 50–58% of bee species (Wood et al. 2023; note this paper was at a global scale). One possible way to address this class imbalance issue is to sub-set the training data so that the proportion of generalists is higher and matches our best guess for what we expect from the data being predicted (as in Elrahman and Abraham 2013).

## Conclusion

Our findings suggest that machine learning models can provide a starting point for predicting specialist and generalist bee species and the phylogenetic, phenological or geographic characteristics related to diet breadth. Identifying bee species’ diet breadths for taxa and for regions where they are unknown can help us answer important questions in bee conservation ecology and plant-pollinator ecology, lead to improved species conservation outcomes, and provide a better understanding of the pollination services that bee species provide.

## Supplementary Information

Below is the link to the electronic supplementary material.

Supplementary file1 (DOCX 625 KB)

Supplementary file2 (CSV 73 KB)

## Data Availability

All code and relevant data files are available on Zenodo: 10.5281/zenodo.8347145.
