# Supplementary material for: Pollen specialist bee species are accurately predicted from visitation, occurrence and phylogenetic data
Source: Oecologia. 2024 Dec 18;207(1):13. doi: 10.1007/s00442-024-05653-5 (PMC11655600; doi:10.1007/s00442-024-05653-5)
Supplement: Supplementary file 1 — Supplementary file1 (DOCX 625 KB) [file 442_2024_5653_MOESM1_ESM.docx]

**Supplemental Information for:**

# Pollen specialist bee species are accurately predicted from visitation, occurrence and phylogenetic data

# Appendix S1: Supporting Methods

## Updating bee taxon names

To update bee taxon names in our datasets, we used the same methods as Chesshire et al. (2023). Specifically, we referred to Table S3 from that paper, which documented the taxon updates they made and then conducted the same updates in our dataset. However, that paper used geographical information to inform a small number of name updates. For example, they assumed specimens of European species, collected in the U.S.A., were misnamed. To be consistent with their approach, we updated these taxon names in our dataset only if our record had geographic coordinates and the coordinates were located inside the contiguous USA. Otherwise, we excluded the record from our analysis. This removed 223 records of five species from the visitation dataset.

## Calculating phylogenetic diversity of plant genera visited

To estimate the phylogenetic diversity of plant genera visited, we used a transformation of Rao’s quadratic entropy, or $\hat{Q}$, following Ricotta and Szeidl (2009):

$$\hat{Q}=\frac{1}{1-\sum_{i,j}^{S} d_{ij}p_{i}p_{j}}$$

Here, $d_{ij}$ is the phylogenetic distance between plant genera *i* and *j*, and $p_{i}$ and $p_{j}$ are their relative frequencies in the bee’s diet. This transformation is a phylogenetic generalization of the inverse Simpson index (Chao et al. 2010, 2014), and hereafter and in the main text we refer to this metric as “phylogenetic Simpson diversity.” The metric takes on a high value when a bee visits many distantly related plant genera, in an even distribution. It takes on a small value when a bee species visits few, closely related plant genera, in a skewed distribution.

To measure the phylogenetic richness of plant genera a bee species visits we also estimated Faith’s phylogenetic diversity, which is the phylogenetic generalization of species richness (Chao et al. 2010, 2014). However, we found that this was strongly correlated with a number of other predictor variables in our model (*r* > 0.7), and we thus excluded it from our final model. We estimated phylogenetic Simpson diversity and Faith’s phylogenetic diversity using the function ‘hill_phylo’ from the R package *hillR* (Li 2018).

### Cross-validation methods

We used three different methods for blocking the data:

1. Random-stratified blocking (baseline): We divided the data into eight folds, maintaining the same proportion of specialists and generalists in each fold as in the larger dataset. The specialists and generalists included in each fold were chosen at random.
2. Spatial blocking: Following the approach used by [Bahn and McGill (2013)](https://www.zotero.org/google-docs/?broken=G8K5xS), we obtained folds for cross-validation by dividing the data spatially into 8 folds, using as our spatial data the median geographic coordinates for each bee species in North America (see Methods: Estimating phylogenetic and geographic predictors). We divided the bee species in half along their latitudinal axis and in quarters along its longitudinal axis, resulting in 8 spatial blocks total. To divide the data in half by latitude we split the data at the median latitude of the data points. To divide the data in quarters by longitude we split the data at the 25%, 50% and 75% percentile longitudes. For the models trained and tested using spatial blocking, we did not include the median latitude or longitude as predictor variables, because we did not want to extrapolate outside the range of the predictor space used to train the model.
3. Phylogenetic blocking: We divided the data into phylogenetic blocks by bee family, apart from the families Melittidae and Colletidae, which we combined into one block. We did this because Melittidae had only three generalist species in our dataset. This resulted in five blocks total. For the models trained and tested using phylogenetic blocking, we did not include any bee phylogenetic predictors in the model, because we did not want to extrapolate outside the range of the predictor space used to train the model.

# Literature cited

Bahn V, McGill BJ (2013) Testing the predictive performance of distribution models. Oikos 122:321–331. https://doi.org/10.1111/j.1600-0706.2012.00299.x

[Bossert S, Wood TJ, Patiny S, et al (2022) Phylogeny, biogeography and diversification of the mining bee family Andrenidae. Syst Entomol 47:283–302. https://doi.org/10.1111/syen.12530](https://www.zotero.org/google-docs/?A6YpsR)

Chao, A., Chiu, C.-H. and Jost, L. 2010. Phylogenetic diversity measures based on Hill numbers. - Phil. Trans. R. Soc. B 365: 3599–3609.

Chao, A., Chiu, C.-H. and Jost, L. 2014. Unifying Species Diversity, Phylogenetic Diversity, Functional Diversity, and Related Similarity and Differentiation Measures Through Hill Numbers. - Annu. Rev. Ecol. Evol. Syst. 45: 297–324.

Chesshire, P. R., Fischer, E. E., Dowdy, N. J., Griswold, T. L., Hughes, A. C., Orr, M. C., Ascher, J. S., Guzman, L. M., Hung, K. J., Cobb, N. S. and McCabe, L. M. 2023. Completeness analysis for over 3000 United States bee species identifies persistent data gap. Ecography e06584. <https://doi.org/10.1111/ecog.06584>

[Freitas (2024) Correction to: UCE phylogenomics, biogeography, and classification of long-horned bees (Hymenoptera: Apidae: Eucerini), with insights on using specimens with extremely degraded DNA. Insect Syst Divers 8:3.](https://www.zotero.org/google-docs/?A6YpsR) <https://doi.org/10.1093/isd/ixae005>

Henríquez-Piskulich P, Hugall AF, Stuart-Fox D (2024) A supermatrix phylogeny of the world’s bees (Hymenoptera: Anthophila). Mol Phylogenet Evol 190:107963. https://doi.org/10.1016/j.ympev.2023.107963

Li, D. 2018. hillR: taxonomic, functional, and phylogenetic diversity and similarity through Hill Numbers. - JOSS 3: 1041.

Ricotta, C. and Szeidl, L. 2009. Diversity partitioning of Rao’s quadratic entropy. - Theoretical Population Biology 76: 299–302.

Russo L (2016) Positive and negative impacts of non-native bee species around the world. insects 7:. https://doi.org/10.3390/insects7040069

Wood TJ, Ghisbain G, Rasmont P, et al (2021) Global patterns in bumble bee pollen collection show phylogenetic conservation of diet. J Anim Ecol 90:2421–2430. https://doi.org/10.1111/1365-2656.13553

# Supporting Tables

**Table S1**. List of non-native bee species in the United States, based on [Russo (2016)](https://www.zotero.org/google-docs/?broken=vw2HKm).

| Bee species |
| --- |
|  |
| Andrena wilkella |
| Anthidium manicatum |
| Anthidium oblongatum |
| Anthophora villosula |
| Apis mellifera |
| Ceratina cobaltina |
| Ceratina dallatorreana |
| Chelostoma campanularum |
| Coelioxys coturnix |
| Euglossa dilemma |
| Halictus tectus |
| Heriades truncorum |
| Hoplitis anthocopoides |
| Hylaeus albonitens |
| Hylaeus hyalinatus |
| Hylaeus leptocephalus |
| Hylaeus punctatus |
| Hylaeus strenuus |
| Hylaeus variegates |
| Lasioglossum eleutherense |
| Lasioglossum leucozonium |
| Lasioglossum zonulum |
| Lithurgus chrysurus |
| Lithurgus scabrosus |
| Megachile apicalis |
| Megachile chlorura |
| Megachile concinna |
| Megachile ericetorum |
| Megachile fullawayi |
| Megachile lanata |
| Megachile rotundata |
| Megachile sculpturalis |
| Osmia caerulescens |
| Osmia cornifrons |
| Osmia taurus |
| Plebia frontalis |
| Xylocopa appendiculata |

**Table S2.** List of sources for the visitation dataset, and the number of records from each.

| **source** | **n** |
| --- | --- |
| A. Thessen. 2014. Species Associations Extracted from EOl Text Data Objects Via Text Mining. | 4156 |
| Arizona State University Hasbrouck Insect Collection | 586 |
| Carril Om, Griswold T, Haefner J, Wilson J.S. (2018) Wild Bees of Grand Staircase-Escalante National Monument: Richness, Abundance, And Spatio-temporal Beta-diversity. PeerJ 6:e5867 https://doi.org/10.7717/peerj.5867 | 4926 |
| Food Webs and Species Interactions in the Biodiversity of UK And Ireland (Online). 2017. Data Provided by Malcolm Storey. Also available from http://bioinfo.org.uk. | 3 |
| Frost Entomological Museum, Pennsylvania State University | 573 |
| Harvard University M, Morris P J (2021). Museum of Comparative Zoology, Harvard University. Museum of Comparative Zoology, Harvard University. | 76 |
| http://inaturalist.org is a Place Where You Can Record What You See in Nature, Meet Other Nature Lovers, and Learn About the Natural World. | 31202 |
| https://mangal.io - The Ecological Interaction Database. | 832 |
| Illinois Natural History Survey Insect Collection | 5 |
| Lamanna, J.A, Burkle, L.A, Belote, R.T, Myers, J.A. Biotic and Abiotic Drivers of Plant Pollinator Community Assembly Across Wildfire Gradients. J Ecol. 2020; https://doi.org/10.1111/1365-2745.13530 . | 1 |
| National Database Plant Pollinators. Center For Plant Conservation at San Diego Zoo Global. Accessed via https://saveplants.org/national-collection/pollinator-search/ On 2020-06-05. | 1684 |
| Natural History Collections Managed By ARCTOS (https://arctosdb.org) Accessed via https://vertnet.org. | 411 |
| Olito, Colin; Fox, Jeremy W. (2015), Data From: Species Traits and Abundances Predict Metrics of Plant Pollinator Network Structure, but not Pairwise Interactions, Dryad, Dataset, https://doi.org/10.5061/dryad.7st32 | 10 |
| Ollerton, J., et al. (2022). Pollinator-Flower Interactions in Gardens During the Covid-19 Pandemic Lockdown of 2020. Journal of Pollination Ecology, 31, 87. https://doi.org/10.26786/1920-7603(2022)695 | 91 |
| Pensoft Darwin Core Archives Available via Integrated Publication Toolkit | 4606 |
| Pensoft Darwin Core Archives with Associate Taxa Columns | 3849 |
| Purdue Entomological Research Collection | 3392 |
| Redhead, J.W.; Coombes, C.F.; Dean, H.J.; Dyer, R.; Oliver, T.H.; Pocock, M.J.O.; Rorke, S.l.; Vanbergen, A.J.; Woodcock, B.A.; Pywell, R.F. (2018). Plant-pollinator Interactions Database for Construction of Potential Networks. NERC Environmental Information Data Centre. https://doi.org/10.5285/6d8d5cb5-bd54-4da7-903a-15bd4bbd531b | 202 |
| Robert L. Minckley San Bernardino Valley from the Year 2000 To 2011. | 476 |
| San Diego Natural History Museum | 8 |
| Sarah E Miller. 12/13/2016. Species Associations Manually Extracted from Onstad, D.W. Edwip: Ecological Database of the World's Insect Pathogens. Champaign, Illinois: Illinois Natural History Survey, [23/11/2016]. http://insectweb.inhs.uiuc.edu/pathogens/edwip. | 50 |
| Sarah E Miller. 5/30/2016. Interactions From Various Papers. | 26 |
| Sarah E Miller. 6/19/2015. Species Associations Manually Extracted from Datasets https://www.nceas.ucsb.edu/interactionweb/resources.html. | 212 |
| Sarah E Miller. 6/22/2015. Species Associations Manually Extracted from Datasets https://www.nceas.ucsb.edu/interactionweb/resources.html. | 224 |
| Sarah E Miller. 6/25/2015. Species Associations Manually Extracted from Robertson, C. 1929. Flowers And Insects: Lists of Visitors to Four Hundred And Fifty-three Flowers. Carlinville, Il, USA, C. Robertson. | 129 |
| Sarah E. Miller. 07/06/2017. Information Extracted from Dataset https://www.idigbio.org/portal/recordsets/db4bb0df-8539-4617-ab5f-eb118aa3126b. | 813 |
| Species Connect. https://speciesconnect.com | 4 |
| Symbiota Collections of Arthropods Network (SCAN) | 53691 |
| Texas A&M University Insect Collection | 212 |
| The Albert J. Cook Arthropod Research Collection | 35 |
| The International Barcode of Life Consortium (2016). International Barcode of Life Project (IBOL). Occurrence Dataset https://doi.org/10.15468/inygc6 | 12 |
| United States Geological Survey (USGS) Pollinator Library. https://www.npwrc.usgs.gov/pollinator. | 162 |
| University of California Santa Barbara Invertebrate Zoology Collection | 75 |
| University of Colorado Museum of Natural History Entomology Collection | 2086 |
| University of Kansas Natural History Museum | 6740 |
| University of Michigan Museum of Zoology Insect Division. Full Database Export 2020-11-20 Provided by Erika Tucker and Barry O’Conner. | 3506 |
| University of New Hampshire Collection of Insects and Other Arthropods UNHC-UNHC | 2489 |
| University of New Hampshire Donald S. Chandler Entomological Collection | 2442 |
| USGS Biodiversity Information Serving Our Nation (BISON) IPT | 19508 |
| Web of Life. http://www.web-of-life.es . | 1375 |

**Table S3**. Taxonomic adjustments to the bee authoritative and visitation datasets based on the Henríquez-Piskulich (2024) phylogeny.

| **Bee genus in datasets** | **Bee genus in Henríquez-Piskulich** | **Reference** |
| --- | --- | --- |
| Pseudopanurgus | Protandrena | Bossert et al. 2022 |
| Peponapis | Xenoglossa | [Freitas et al. 2023](https://www.zotero.org/google-docs/?broken=cxrcg8) |
| Tetraloniella | Xenoglossa | [Freitas et al. 2023](https://www.zotero.org/google-docs/?broken=1p5CMU) |
| Syntrichalonia | Xenoglossa | [Freitas et al. 2023](https://www.zotero.org/google-docs/?broken=GwmZcP) |
| Cemolobus | Xenoglossa | [Freitas et al. 2023](https://www.zotero.org/google-docs/?broken=xKP10D) |
| Micralictoides | Dufourea | [Timberlake 1939](https://www.zotero.org/google-docs/?broken=mcTpHf) |

**Table S4.** Bee species with at least 200 records in the visitation dataset, with less than half to their putative pollen host. Listed are the bee species, their host plants, the host plant rank (genus or family), the proportion of visits to the host plant in the visitation dataset, and the bee’s sample size in the visitation dataset. Bee species are ordered by sample size.

| **Bee** | **Host** | **Host rank** | **Proportion of visits to host** | **Sample size** |
| --- | --- | --- | --- | --- |
| *Protoxaea gloriosa* | Kallstroemia | genus | 0.04 | 1366 |
| *Megachile brevis* | Asteraceae | family | 0.31 | 1148 |
| *Megachile mendica* | Asteraceae | family | 0.34 | 934 |
| *Megachile policaris* | Helianthus | genus | 0.03 | 673 |
| *Calliopsis andreniformis* | Trifolium | genus | 0.06 | 519 |
| *Augochloropsis metallica* | Vaccinium | genus | 0.01 | 447 |
| *Anthophora terminalis* | Lamiaceae | family | 0.17 | 442 |
| *Lasioglossum quebecense* | Vaccinium | genus | 0.14 | 344 |
| *Perdita knowltoni* | Chrysothamnus | genus | 0.24 | 309 |
| *Andrena sigmundi* | Salix | genus | 0.46 | 255 |
| *Hesperapis pellucida* | Eschscholzia | genus | 0.28 | 212 |

**Table S5**. Specialist bee species for which our random forest models provide a specialist classification probability of less than 50%. Specialist probability is defined here as the mean out-of-bag, specialist classification rate in the spatially validated random forest models.

| **Specialist name** | **Specialist probability** |
| --- | --- |
| Lasioglossum quebecense | 0.10 |
| Lasioglossum oceanicum | 0.13 |
| Andrena tridens | 0.14 |
| Andrena melanochroa | 0.17 |
| Megachile brevis | 0.19 |
| Hylaeus basalis | 0.20 |
| Anthophora terminalis | 0.21 |
| Calliopsis andreniformis | 0.21 |
| Megachile melanophaea | 0.22 |
| Augochloropsis metallica | 0.23 |
| Hylaeus verticalis | 0.23 |
| Andrena personata | 0.24 |
| Megachile frigida | 0.26 |
| Megachile mendica | 0.26 |
| Andrena clarkella | 0.28 |
| Megachile pugnata | 0.29 |
| Osmia albolateralis | 0.33 |
| Andrena nigrae | 0.35 |
| Lasioglossum nelumbonis | 0.35 |
| Hylaeus annulatus | 0.36 |
| Osmia georgica | 0.37 |
| Osmia nigrifrons | 0.37 |
| Andrena bradleyi | 0.44 |
| Andrena piperi | 0.44 |
| Andrena sigmundi | 0.44 |
| Megachile policaris | 0.44 |
| Megachile lapponica | 0.47 |
| Osmia coloradensis | 0.47 |
| Osmia integra | 0.48 |

**Table S6.** Generalist bee species for which our random forest models provide a generalist classification probability of less than 50%. Generalist probability is defined here as the mean out-of-bag, generalist classification rate in the phylogenetically-validated random forest models

| **Generalist name** | **Generalist probability** |
| --- | --- |
| Chelostoma cockerelli | 0.02 |
| Hoplitis biscutellae | 0.02 |
| Perdita electa | 0.02 |
| Andrena helianthiformis | 0.03 |
| Andrena dolomellea | 0.04 |
| Panurginus occidentalis | 0.04 |
| Hesperapis trochanterata | 0.05 |
| Ptiloglossa arizonensis | 0.05 |
| Ashmeadiella australis | 0.06 |
| Melissodes robustior | 0.06 |
| Chelostoma philadelphi | 0.07 |
| Perdita bequaerti | 0.07 |
| Ancyloscelis apiformis | 0.08 |
| Colletes stepheni | 0.08 |
| Andrena accepta | 0.09 |
| Andrena faceta | 0.09 |
| Chelostoma incisulum | 0.09 |
| Lasioglossum pallidellum | 0.09 |
| Andrena helianthi | 0.12 |
| Chelostoma rapunculi | 0.12 |
| Lasioglossum pectinatum | 0.12 |
| Osmia sculleni | 0.12 |
| Perdita diversa | 0.12 |
| Andrena anograe | 0.13 |
| Andrena pulverea | 0.13 |
| Diadasia rinconis | 0.13 |
| Andrena astragali | 0.14 |
| Andrena lawrencei | 0.15 |
| Perdita perpallida | 0.15 |
| Hesperapis arida | 0.17 |
| Andrena vicinoides | 0.18 |
| Megachile pusilla | 0.18 |
| Hesperapis ilicifoliae | 0.19 |
| Lasioglossum subversans | 0.19 |
| Ptiloglossa jonesi | 0.19 |
| Megachile inermis | 0.21 |
| Megachile mucida | 0.21 |
| Lasioglossum cattellae | 0.22 |
| Colletes hyalinus | 0.25 |
| Agapostemon femoratus | 0.27 |
| Andrena mellea | 0.27 |
| Hoplitis robusta | 0.27 |
| Andrena geranii | 0.28 |
| Hylaeus pictipes | 0.28 |
| Melissodes agilis | 0.28 |
| Habropoda laboriosa | 0.30 |
| Lasioglossum synthyridis | 0.30 |
| Andrena banksi | 0.32 |
| Plebeia emerina | 0.32 |
| Anthidium tenuiflorae | 0.33 |
| Anthophora occidentalis | 0.34 |
| Megachile dentitarsus | 0.35 |
| Andrena alleghaniensis | 0.41 |
| Eucera frater | 0.42 |
| Lasioglossum fuscipenne | 0.43 |
| Megachile perihirta | 0.43 |
| Andrena robertsonii | 0.44 |
| Megachile circumcincta | 0.44 |
| Andrena wheeleri | 0.45 |
| Lasioglossum gotham | 0.45 |
| Osmia juxta | 0.45 |
| Lasioglossum nigroviride | 0.46 |
| Anthophora walshii | 0.48 |
| Hylaeus nelumbonis | 0.48 |

**Table S7**. List of predictor variables and their mean importance across model runs. Variable importance is defined as the standardized decrease in the out-of-bag classification rate between the observed value and null expectation for that variable.

| Predictor variable | Mean importance |
| --- | --- |
| Plant phylogenetic diversity | 0.05 |
| Plant Simpson diversity | 0.03 |
| Plant genus identity (2nd eigenvalue) | 0.02 |
| Area (ha) | 0.02 |
| Regional abundance | 0.02 |
| Median latitude | 0.02 |
| Plant family identity (2nd eigenvalue) | 0.01 |
| Plant family identity (1st eigenvalue) | 0.01 |
| Flight season | 0.01 |
| Plant genus identity (1st eigenvalue) | 0.01 |
| Median day of activity | 0.01 |
| Median longitude | 0.01 |
| Agapostemon | 0.01 |
| Halictus | 0.01 |
| Lasioglossum | 0.01 |
| Augochlorella | 0.00 |
| Augochloropsis | 0.00 |
| Augochlora | 0.00 |
| Bombus | 0.00 |
| Plebeia | 0.00 |
| Perdita | 0.00 |
| Macrotera | 0.00 |
| Centris | 0.00 |
| Protoxaea | 0.00 |
| Dieunomia | 0.00 |
| Calliopsis | 0.00 |
| Chelostoma | 0.00 |
| Osmia | 0.00 |
| Protandrena | 0.00 |
| Atoposmia | 0.00 |
| Panurginus | 0.00 |
| Heriades | 0.00 |
| Ashmeadiella | 0.00 |
| Hoplitis | 0.00 |
| Hylaeus | 0.00 |
| Svastra | 0.00 |
| Melissodes | 0.00 |
| Xenoglossa | 0.00 |
| Eucera | 0.00 |
| Anthophorula | 0.00 |
| Colletes | 0.00 |
| Melitoma | 0.00 |
| Florilegus | 0.00 |
| Ptilothrix | 0.00 |
| Diadasia | 0.00 |
| Xylocopa | 0.00 |
| Ceratina | 0.00 |
| Ptiloglossa | 0.00 |
| Eulonchopria | 0.00 |
| Andrena | 0.00 |
| Megachile | 0.00 |
| Sphecodosoma | 0.00 |
| Conanthalictus | 0.00 |
| Megandrena | 0.00 |
| Ancyloscelis | 0.00 |
| Protodufourea | 0.00 |
| Xeralictus | 0.00 |
| Ancylandrena | 0.00 |
| Dufourea | 0.00 |
| Dianthidium | 0.00 |
| Paranthidium | 0.00 |
| Anthidium | 0.00 |
| Trachusa | 0.00 |
| Habropoda | 0.00 |
| Anthophora | 0.00 |
| Anthidiellum | 0.00 |
| Lithurgopsis | 0.00 |
| Melitta | 0.00 |
| Macropis | 0.00 |
| Hesperapis | 0.00 |

# Figures
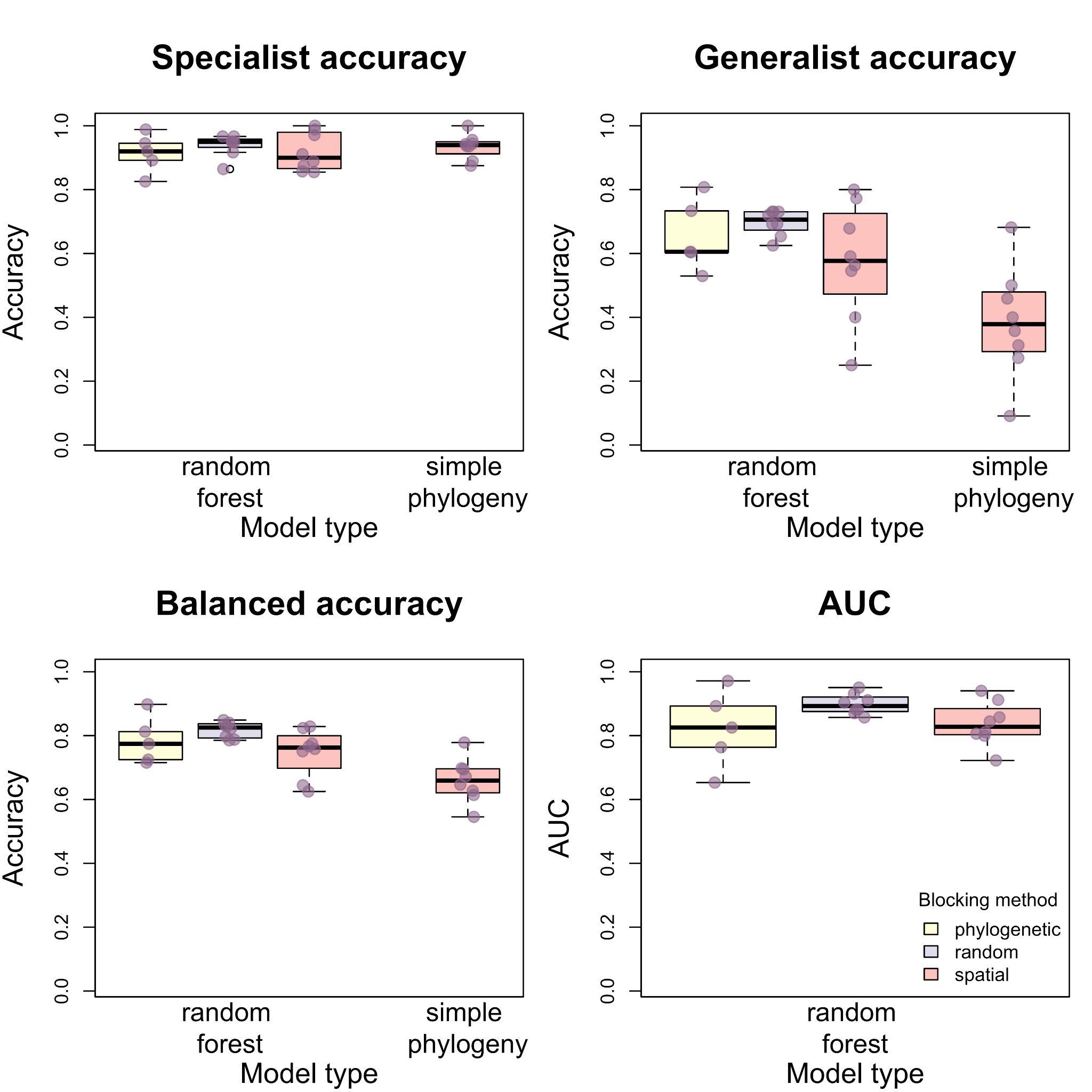


**Figure S1**. Boxplots and data showing estimates of model performance for three different blocking methods. Purple points represent the data, with a random jitter added to allow overlapping points to be seen. Models tested using random stratified blocking (purple boxplots) performed similarly to models tested using spatial and phylogenetic blocking (red and yellow boxplots).


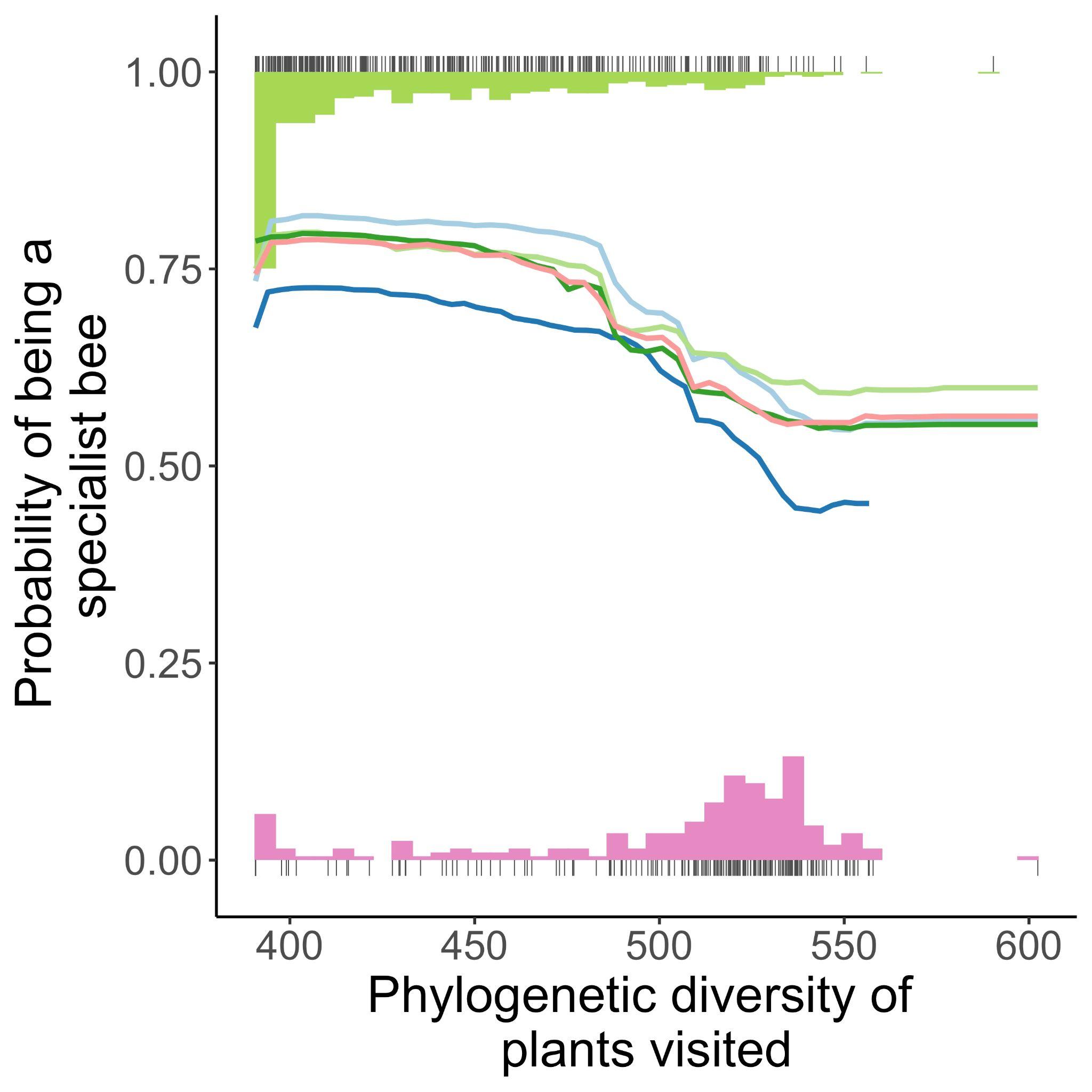


**Figure S2.** Partial dependence plots and data showing the relationship between model predictors and the probability a bee species is a specialist, for the two most important predictor variables in our analysis. The lines show partial dependence plots, with separate colors for each model run (for phylogenetically-blocked models). For the partial dependence plots, predictions are made from the model n x m times, where n is the number of data points and m is an evenly spaced sequence of the predictor variable of interest. All other covariates are held at their true values. The tick points show the data, with tick points at one representing specialist bee species, and tick points at zero representing generalists. The histograms depict the distribution of the tick points.


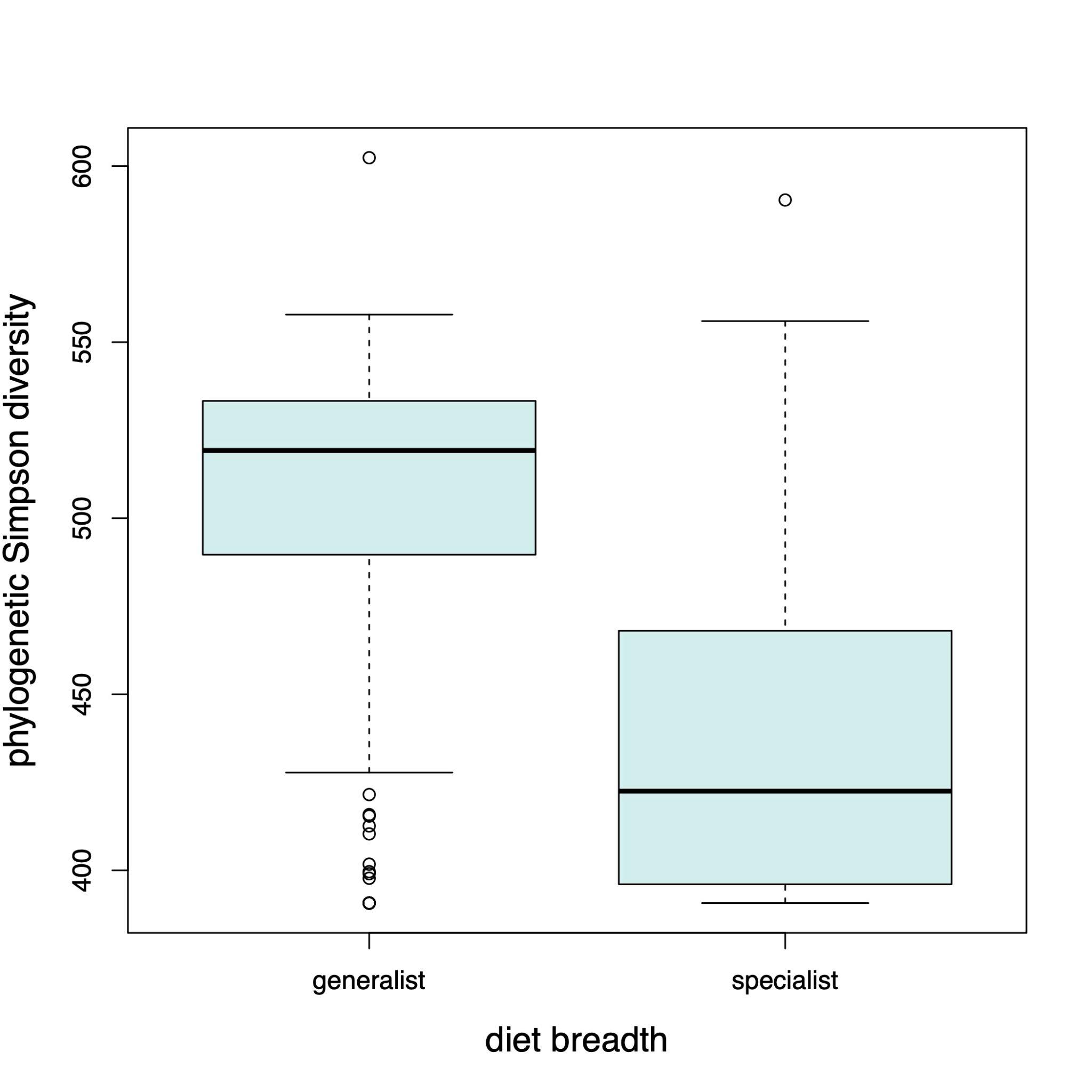


**Figure S3.** Boxplots showing the difference between specialist and generalist bees in the phylogenetic Simpson diversity of plant genera visited. The boxes encompass the first and third quartiles of the data and the thick black line is the median. Plot whiskers extend to 1.5 times the interquartile range.
